# An incidental danger: Left ventricular thrombus in takotsubo syndrome

**DOI:** 10.21542/gcsp.2023.22

**Published:** 2023-08-01

**Authors:** Taylor Beckmann, Hesham Afify, Jishanth Mattumpuram

**Affiliations:** Division of Cardiology, University of Louisville, Louisville, KY, USA

## Abstract

Takotsubo cardiomyopathy is a potentially lethal condition characterized by transient regional systolic dysfunction in the absence of coronary artery ischemia. This syndrome predominantly affects postmenopausal women and is often preceded by physical or emotional stress and often presents with symptoms of acute coronary syndrome, chest pain, and shortness of breath. Although the effects can be transient, takotsubo cardiomyopathy still results in an 8–12% rate of in-hospital mortality, with cardiogenic shock being the most common cause of death. There are known risk factors that increase the likelihood of a patient developing a left ventricular thrombus during the clinical course. The management of these cases is discussed in this report.

## Background

Takotsubo cardiomyopathy (TCM), also known as stress cardiomyopathy, apical ballooning syndrome, or broken heart syndrome, is characterized by transient systolic dysfunction in the apical or mid-segments of the left ventricle (LV) in the absence of coronary artery obstruction. LV dysfunction and loss of contractility can precipitate the symptoms of acute systolic heart failure. This condition is typically triggered by physical or emotional stress, which results in transient myocardial stunning^1^. The postulated mechanisms in which this takes place include excessive catecholamine release, coronary artery spasm, and an exaggerated sympathetic response^2^.

LV thrombi occur in any condition that promotes blood stasis in the ventricle, such as acute myocardial infarction, heart failure with reduced ejection fraction, and acute TCM. The presence of LV thrombi is associated with an increased risk of major adverse cardiovascular events, and 17% of patients with LV thrombi will eventually experience a stroke^3^. This report presents the case of a 43-year-old female incidentally diagnosed with TCM with a left apical thrombus. The patient was managed with oral anticoagulation for three months with ECHO surveillance and follow-up.

## Case

**Figure 1.**
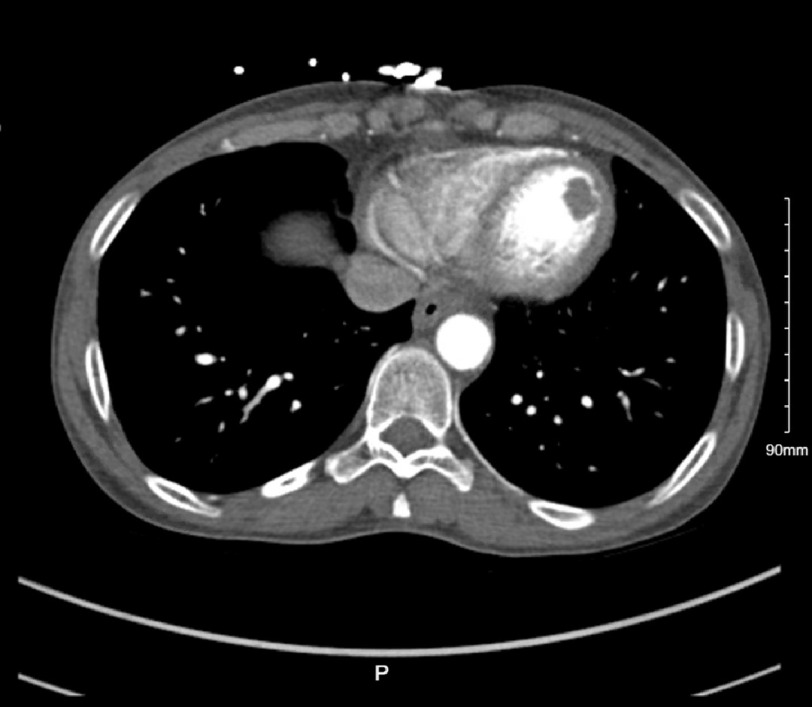
A hypoattenuating lesion (1.7 × 1.3 cm) within the left ventricle concerning a thrombus or mass.

A 43-year-old female with history of anxiety and depression presented to an outside hospital with a primary complaint of severe abdominal pain, nausea, and vomiting. The patient’s initial WBC and lactic acid levels were elevated. High-sensitivity troponin levels were elevated to 390 ng/L on admission and subsequently decreased during the patient’s stay. Other pertinent laboratory values include an elevated BNP level of 1826 pg/mL and an elevated CRP level of 22.07 mg/L. Her initial EKG showed new T-wave inversions in leads II, III, V4-6, and QT prolongation, with a QTC of 532 ms. The patient was given fluids, and a computed tomography (CT) scan of the abdomen/pelvis was performed (Figure 1), which showed an incidental hypoattenuating lesion (1.7 × 1.3 cm within the left ventricle concerning a thrombus or mass.

**Figure 2.**
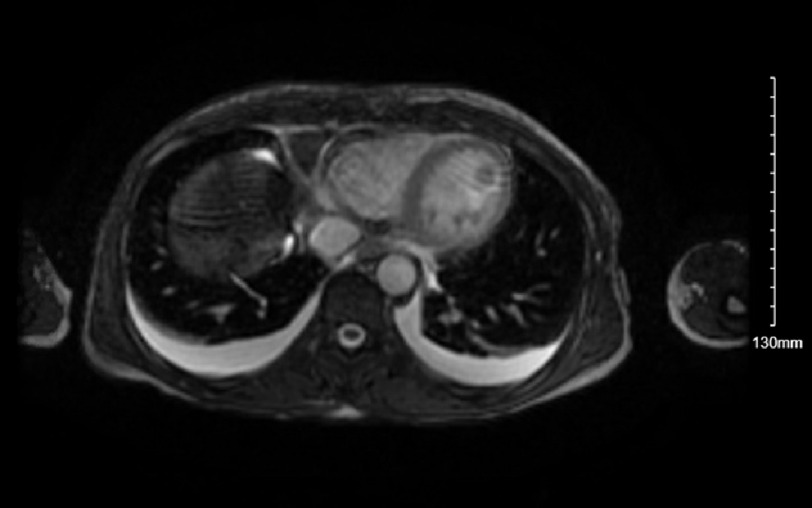
Axial view CMR view 16 × 14 mm spherical mass is seen in the apical inferior LV cavity, which is likely a thrombus based on imaging features (avascular, no gadolinium uptake, no LGE on delayed imaging and severe apical hypokinesis).

**Figure 3.**
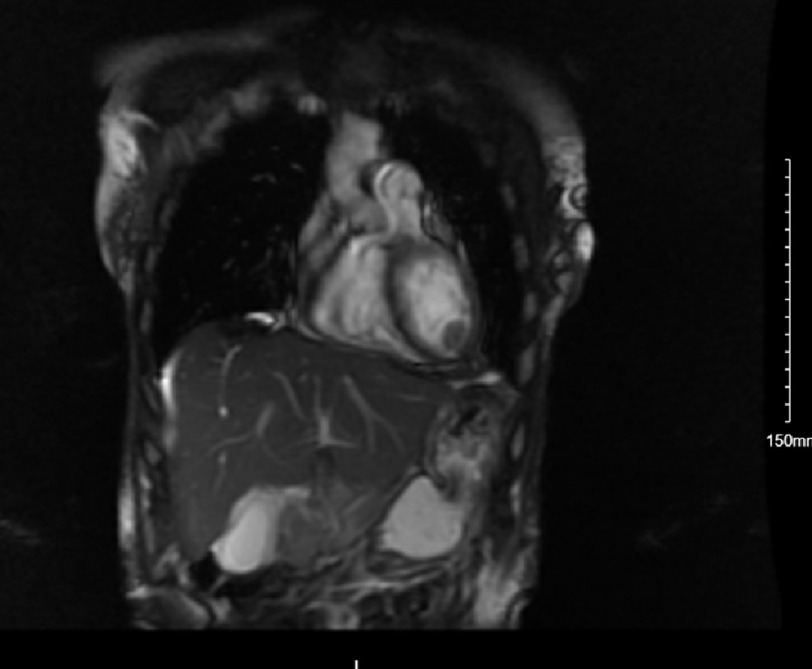
Coronal CMR 16 × 14 mm spherical mass is seen in the apical inferior LV cavity.

She became hypotensive after admission, was placed on vasopressor support for undifferentiated shock, and was then transferred to a tertiary care center ICU. Over the next 24 h, the patient’s shock resolved with a negative sepsis workup. A 2D echocardiogram with contrast revealed an LVEF of 40% and a 1.6 cm mobile LV thrombus with a high embolic risk. Other transthoracic echocardiogram (TTE) findings include an akinetic apex and severe hypokinesis of the mid LV segments with preserved function at the base. Given the patient’s reduced ejection fraction, her presentation of shock was retrospectively labeled cardiogenic shock. Cardiac MRI was then performed (Figures 2 & 3) and showed a takotsubo cardiomyopathy pattern in the LV with an LVEF of 33% and a 16 × 14 mm apical mass that was avascular and likely a thrombus. The patient was started on a heparin drip. After the heparin drip was discontinued, the patient transitioned to 3-month course of apixaban at discharge. She was initiated on goal-directed medical therapy for heart failure with reduced ejection fraction and scheduled for cardiology follow-up.

## Discussion

This patient initially presented with abdominal pain, but was found to have an incidental LV mass, elevated troponins, non-specific EKG changes for ischemia, and a low EF. Given the lack of chest pain and the nature of presentation (young age, lack of cardiac history, or risk factors), the suspicion of ACS was low. The patient’s presentation was consistent with TCM based on diagnostic criteria from the international takotsubo syndrome diagnostic criteria published in 2014 (the InterTAK diagnostic criteria)^4^. The criteria include transient left ventricular dysfunction presenting as apical ballooning or midventricular, basal, or focal wall-motion anomalies; an emotional, physical, or combined trigger may precede the disease onset (but is not obligatory); neurological disorders, as well as pheochromocytoma, may serve as triggers; new electrocardiographic anomalies are present (e.g., ST-segment elevation, ST-segment depression, T wave inversion, and QTc prolongation); moderate elevation in cardiac biomarker levels (e.g., troponin and creatine kinase) and significant elevation of brain natriuretic peptide; no evidence of myocarditis; and significant coronary artery disease may coexist^4^.

Of these criteria, our patient presented with physical stress (possible gastritis) with LV dysfunction in an apical ballooning pattern with EKG changes and elevated troponins without the presence of myocarditis. This case is unique in that TCM was an incidental finding and did not cause symptoms; the majority of TCM cases are more symptomatic. Although this patient lacked specific cardiac symptoms, the presence of the LV thrombus and the threat of embolization warranted close surveillance until resolution of both the thrombus and return of cardiac function.

## Epidemiology

TCM is a poorly understood phenomenon that is difficult to diagnose and treat. TCM mostly occurs in females (87.5%) and most commonly occurs in the age range of 60–69^5^. Rough estimates gauge that there are approximately 15–30 cases per 100,000 people per year^4,6^. Although not present in this patient, other risk factors predisposing to TCM include diabetes mellitus, which is present in 10–25% of cases, asthma, and cannabis use disorder^3^. Physical or emotional stress were found to be the precipitating stressors in approximately 50% of cases according to 2015 review of 250 cases^5^.

TCM is often initially mislabeled as acute coronary syndrome on presentation, as they share presenting symptoms and findings such as chest pain, ST-changes on EKG, elevated troponins, and pro-BNP. In fact, before 2019 the incidence of stress cardiomyopathy was observed in approximately 1–2% of patients presenting with ACS (later determined to be TCM)^7^. The incidence of TCM has been reported to increase since the COVID-19 pandemic, and Jabri et al. reported an incidence of TCM in 2020 of 7–8% of ACS presentations^8^. This number does not account for incidentally found TCM cases that were not labeled as ACS on presentation. The increase in incidence has been attributed to both increased stress and disaster-related fear in the community following the COVID-19 pandemic, along with manifestations of COVID-19 disease^9,10^. It is unclear how much each of these factors contributes to the increased incidence; however, a study performed by the European Society of Cardiovascular Imaging examining echocardiography findings in 1216 COVID-19 patients in 2020 found a TCM incidence of 2% in this population, far above the 15–30 cases of 100,00 people per year in the general population^1,9–11^. More data is needed to draw accurate conclusions regarding the changing incidence of TCM.

## Pathophysiology

The exact pathophysiology of TCM remains a mystery, although several mechanisms have been proposed. Observational imaging studies have shown that patients in the acute phase of TCM have altered cerebral blood flow compared with controls^12^. This may represent a complex stress response pathway in the neocortical and limbic areas through activation of brainstem adrenergic neruons^12,13^. This would occur through the release of large amounts of norepinephrine and neuropeptide y (NPY) through the autonomic nervous system at the level of the myocardium, causing a direct toxic effect. Patients with TCM are known to have increased plasma levels of stress hormones and catecholamines.

Wittstein et al. showed that TCM patients in the acute phase have higher plasma catecholamines than even STEMI patients^2^. A 2012 study injected large concentrations of epinephrine into rats and observed a reversible apical ballooning pattern in the LV with spared basilar contractility. Interestingly, this did not occur with large injections of norepinephrine^14^. This is thought to occur because epinephrine recruits and switches beta adrenergic receptors (B-AR) on the myocardium from a positively ionotropic G-protein-coupled receptor(G_s_) to a negatively ionotropic G-protein-coupled receptor(G_i_)^14^. As the serum epinephrine levels fell in this same experiment, B-ARs were internalized and replaced with new receptors that were uncoupled to Gi stimulus, restoring normal function to the remaining cardiomyocytes^14^. A different theory postulates that coronary vasospasm may be at play in the mechanism of TCM, but the finding that large doses of norepinephrine are unable to initiate apical dysfunction has lead some to doubt this idea^1,1,14^.

A prospective study by Angelini showed that the extensive coronary artery spasm that produced LV apical ballooning in four patients during acetylcholine testing in the cath lab has, at the very least, provided evidence otherwise^15^.

Another theory states that impaired cardiac function is a result of impaired microvascular perfusion, leading to a demand-supply mismatch and ischemic myocardial stunning^16^. A 2010 study performed myocardial perfusion scans in patients during the acute phase of TCM and at 1 month follow up. These findings were compared to those of the same test run in STEMI patients and showed reversible microvascular dysfunction in the acute TCM phase that was reversed after 1 month^17^. Whether all these mechanisms occur simultaneously or whether they represent separate phenotypes of this disease is yet to be determined.

## Imaging

Stress cardiomyopathy is often initially diagnosed using echocardiography, and certain echocardiographic findings seen on echo can help differentiate TCM from ACS. There are multiple morphologies of TCM, the typical, or apical, ballooning pattern with apical akinesis or “octopus pot” is seen in approximately 80% of cases and can help diagnose TCM with the appropriate set of matching findings^18^.

Some atypical morphologies include the midventricular ballooning pattern present in 10–20% of cases and is often associated with more severe hemodynamic compromise, a basilar stunning pattern, severe biventricular stunning, and more focal stunning patterns.nts^19^. In this case, an akinetic apex and severe hypokinesis of the mid-LV segments were visualized on TTE along with a 1.6 cm thrombus. The sensitivity of TTE for detecting left ventricular thrombosis is 92%, with a specificity of 86%, depending on the patient windows and image quality^20,21^. Cardiac MRI (CMR) was used here to better characterize the LV mass and investigate the extent of LV dysfunction. CMR has the added advantage of being able to identify the presence of reversible and irreversible myocardial damage such as scarring and myocardial edema^22^. TTE remains the most common imaging modality used for the evaluation of TCM, even though intracardiac thrombi can be under-recognized owing to variable image quality. CMR has a sensitivity and specificity of 92% and 99%, respectively, for the detection of ventricular thrombi, especially in early gadolinium sequences, and provides more information about the myocardium^23,24^. CMR can also visualize areas of myocardial edema, inflammation, and scarring that are not seen on TTE. These characteristics can help differentiate stress cardiomyopathy, myocarditis, ACS and other pathology^20^. Of note, computed tomography first detected a left ventricular “mass suspicious for thrombus” and required TTE and CMR imaging for confirmation.

LV thrombi is predicted to occur in 2.2%−8.9% of TCM cases^3^. Of the cases where LV thrombi do form, 17–30% of patients may develop embolic strokes^3^. Some proposed mechanisms of clot formation include; dysfunctional contraction of the LV apical region causing transient apical aneurysm and hemostasis, endocardial injury with release of thrombogenic materials, and catecholamine induced platelet aggregation^1,23,25^. Surges in catecholamines are known to induce changes to red blood cells through ion exchanges that cause red blood cell swelling and increased cell membrane rigidity that promotes red blood cell aggregation and clots^1^.

Risk factors that are known to increase the likelihood of LV thrombus formation in TCM include the presence of an apical ballooning pattern of the LV, elevated CRP or WBC, and increased troponin I levels (>10 ng/mL)^3,26^. Elevated troponin levels often correspond to the level of myocardial injury, and higher levels indicate a larger area of stunned myocardium^1,13^. The typical apical ballooning form of TCM seems to be particularly vulnerable to ventricular thrombus formation, likely owing to the larger amount of myocardium affected^18,23^. Cytokines released from WBCs and other inflammatory markers can promote thrombus formation. Elevated WBC and CRP have also been linked to increase risk of thrombus in acute myocardial infarction^3,13,18^. All these risk factors were present in this case. TCM itself has also been shown to be an independent risk factor for thrombus formation, as the coagulation system itself becomes altered. Multiple endothelial and clotting activation (plasminogen and von Willebrand factor) markers have been shown to be markedly elevated in patients with TCM compared to those in a healthy population, according to a 2016 study of 150 women^25,27^.

After an initial diagnosis, repeat imaging is performed to ensure the resolution of LV function. As far as LV thrombus surveillance is concerned, there are currently no recommendations in TCM. A registry of 541 patients showed that the majority of LV thrombi will form on day 1–5 after a stressor in the acute phase of TCM, although delayed formation has also been noted in many case studies^3,24^. The left ventricular dysfunction attributed to TCM is reliably resolved by 3 months and follow up cardiac imaging for resolution has been recommended at this time^19,27,28^. The 541 patient cohort from the German Italian Stress Cardiomyopathy registry estimated the rate of thromboembolic events in the first 30 days to be 1.3%^3^. A separate registry of 400 patients admitted to a large US tertiary care center with a primary diagnosis of TCM reported a thromboembolic rate of 8.9%^26,29^. The 2014 American College of Cardiology (ACC) guidelines provide a 2b class C recommendation for the prophylactic use of anticoagulation to prevent LV thrombi in stress cardiomyopathy^16^. This recommendation was given before the COVID-19 pandemic, and before the incidence of TCM increased.

These recommendations also do not consider the risk factors for the development of LV thrombi in TCM. No updated recommendations have been made by the ACC on this topic. A systematic review of 26 trials suggested that anticoagulation should be started at the time of diagnosis with or without the presence of an LV thrombus and continued for 10 days, especially in those with risk factors for clot formation^30,31^. With a known LV thrombus, anticoagulation is recommended as a class 1c recommendation, according to the 2014 ACC guidelines. Although the time course for anticoagulation was not given in these guidelines for TCM, the recommendations for anticoagulation of LV thrombi in post-MI patients are for 3 months per the 2023 ACC guidelines and can be reasonably applied here. Observational studies with intravenous heparin transitioned to warfarin have shown reduced rates of strokes^3^. Oral direct anticoagulants (DOACs) have been shown to be as effective as warfarin in the treatment of LV thrombi of any cause according to a review of two meta-analysis by Honan et al. ^32,33^. Heparin and apixaban were the agents chosen in this case with 3 month follow up to re-evaluate cardiac imaging and termination of anticoagulation.

## What have we learned?

This patient had a follow-up TTE performed 8 weeks after the initial LV thrombus was discovered, which showed a recovered EF of 50% and no evidence of LV thrombus. At the next cardiology follow-up appointment at 5 months, anticoagulation therapy was discontinued, but the patient was kept on GDMT. She had no issues with medications and tolerated GDMT; therefore, the cardiac follow-up was broadened to 1 year.

Although specific management strategies for takotsubo syndrome lack RCT data, more retrospective data continue to arise to expand our understanding of the management of TCM and to recognize those who will form LV thrombi. The mainstay of diagnosis in TCM is echocardiography, but Cardiac MRI is superior in providing more detail and can detect LV thrombi that are not seen on TTE. Patients diagnosed with TCM with an LV thrombus or with multiple risk factors for the formation of an LV thrombus (elevated troponin I <10 ng/mL, elevated CRP or WBC, and apical ballooning pattern on imaging) would benefit from anticoagulation for three months to reduce the risk of thromboembolic events.

Patients without these risk factors for thrombus formation may also benefit from anticoagulation for at least 10 days and up to 3 months. If patients are not on anticoagulation, interval imaging may be prudent to screen for left ventricular thrombi, as TCM promotes clot formation. Although warfarin has been more well studied for this issue, recent data on DOACs are just as effective in the setting of LV thrombi. Follow-up cardiac imaging is recommended within 3 months to ensure the return of cardiac function and resolution of any thrombi. Given the lack of symptoms at presentation, managing physicians should have a high suspicion for TCM based on demographics and risk factors to identify these incidental cases.
